# Mechanical Thrombectomy for a Clot in Transit With Adherence to the Tricuspid Valve

**DOI:** 10.7759/cureus.46636

**Published:** 2023-10-07

**Authors:** Kang Woo Kim, Mareril Wheeler, Franklin Schneider, Gerardo Carino

**Affiliations:** 1 Internal Medicine, Brown University, Providence, USA; 2 Pulmonary and Critical Care, Brown University, Providence, USA; 3 Cardiology, Brown University, Providence, USA

**Keywords:** reperfusion therapy, right heart thrombus, mechanical thrombectomy (mt), submassive pulmonary embolism, clot in transit

## Abstract

This case report investigates the management of a clot in transit (CIT), a rare but possibly life-threatening condition discovered in a small percentage of pulmonary embolism (PE) cases. CITs are thrombi lodged within the right-side heart chambers or the major veins, and there are currently no universal guidelines for their management though the literature has shown reduced mortality with reperfusion therapy compared to anticoagulation alone. In this case, a 96-year-old male who presented with a submassive PE was initially stabilized with anticoagulation and was then discovered to have a CIT with adherence to the tricuspid valve. The patient underwent a successful mechanical thrombectomy using the Inari FlowTriever (Inari Medical, Irvine, CA), an FDA-approved device for CIT removal. Overall, this manuscript supports this percutaneous intervention in intermediate to high-risk PE patients with concomitant CIT, offering an alternative to thrombolysis and cardiothoracic surgery, which carry their own risks. Furthermore, the unique characteristic of the CIT in this patient suggests a potential for further investigation into the diversity of CIT morphology and its significance.

## Introduction

A clot in transit (CIT), also known as a right heart thrombus, is a thrombus found inside the right-side chambers of the heart, or the superior or inferior vena cava. Found in approximately 4% of patients suffering from a pulmonary embolism (PE), a CIT is typically discovered during echocardiography [1,2]. CITs have three categories based on appearance. Type A is the most common and appears hypermobile and serpentine. This form of CIT originates from a deep vein thrombus. These clots pose the highest risk of embolization into the pulmonary circuit. Type B appears broad-based and globular and is typically immobile with adherence to the chamber wall. Lastly, type C shares features of both A and B, being broad-based and hypermobile, though they are uncommon in the literature [2,3]. While CITs have a significant association with increased short-term all-cause mortality and PE-related deaths, no universal treatment guideline for CITs exists, and it is important to evaluate the management and success of individual cases.

## Case presentation

A 96-year-old male with a significant past medical history, including prostate cancer treated with radiation and androgen ablation, deep vein thrombosis of the right proximal peroneal vein, and superficial thrombophlebitis of the right great saphenous vein in 2021 (treated with six months of apixaban therapy) presented to the hospital with shortness of breath. On examination, the patient was tachycardic, normotensive, and saturating at 90% with 4 L/min oxygen via nasal cannula. He was not on anticoagulation at the time of his presentation. Notable labs are provided below (Table 1).

**Table 1:** Pertinent lab results on admission

Laboratory Test	Ref Range & Units	Patient Result
B-type natriuretic peptide	0.0 – 176 pg/mL	1022.7
High-sensitivity troponin	3 – 57 ng/L	83
D-dimer	0 – 230 ng/mL	2525
pH, venous	7.32 – 7.42	7.4
pCO2, venous	42 – 50 mmHg	42

**Figure 1:**
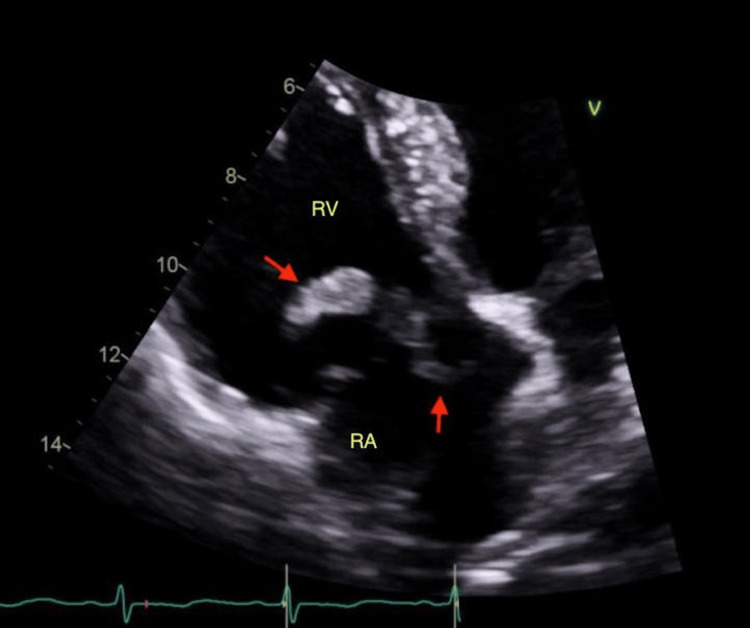
Transthoracic echocardiogram of the clot in transit Apical four-chamber view: this image reveals thickened tricuspid leaflets with two mobile echo densities seen on the atrial and ventricular valve surfaces (indicated with arrows). RA: right atrium; RV: right ventricle

The electrocardiogram showed no ST elevations. Computed tomography angiography (CTA) PE demonstrated saddle PE with bilateral upper and lower lobar extension. Enlargement of the patient’s right ventricle (RV) on CTA suggested right-sided cardiac strain. The patient’s calculated pulmonary embolism severity index (PESI) score was elevated at 176, indicating very high risk with 10 to 24.5% 30-day mortality in this group. The patient was admitted to the medical intensive care unit for the management of a submassive PE and was started on an IV heparin drip. By hospital day two, the patient was saturating well on room air with vitals within normal limits, with a plan to transfer to the floor level of care. However, a routine echocardiogram performed that morning revealed a moderately dilated RV size with moderately reduced systolic function consistent with RV strain. Calculated pulmonary artery (PA) systolic pressure was moderately elevated (59 mmHg). Thickened tricuspid leaflets with two mobile echo densities were identified on the atrial and ventricular valve surfaces, suggestive of CIT (Figure 1).

At this point, we discussed the case with our hospital’s PE response team, which consisted of physicians from the intensive care unit, interventional radiology, interventional cardiology, and cardiothoracic surgery, and the patient underwent a successful aspiration thrombectomy of right atrial/ventricular thrombi under TTE guidance followed by aspiration thrombectomy of bilateral pulmonary arteries. Heparin was continued perioperatively. The procedures were performed using an Amplatz Super Stiff Straight Tip Short Taper 1 cm (Boston Scientific, Marlborough, MA), a Flowsaver Blood System (Inari Medical, Irvine, CA), an Initri24 Introducer Sheath (Inari Medical), and an Inari Triever 24Fr (Inari Medical). Post-procedure, the patient experienced no complications. Hospital discharge occurred on hospital day four with the patient anticoagulated on apixaban.

## Discussion

Several studies have highlighted the increased morbidity and mortality associated with the finding of a CIT. The International Cooperative Pulmonary Embolism Registry studied 2,454 patients from 1995 to 1996 with PE and found CIT in 4% of the patients. In patients treated with heparin alone, significantly higher 14-day mortality occurred in patients with CIT compared to those without (23.5% vs 8%) [4]. On average, patients with CIT were more often found to be hemodynamically compromised, with lower systolic blood pressures, higher heart rates, and more extensive right ventricle hypokinesis. The risks of CIT were further highlighted in one of the largest meta-analyses to date; when adjusting for baseline clinical and echocardiographic variables, patients with CIT were concluded to present a therapeutic emergency even when hemodynamically stable due to risks of further embolization [1]. Furthermore, patients with CIT had double the frequency of right ventricular hypokinesis, and more frequent rates of hypotension, tachycardia, hypoxemia, syncope, and an elevated troponin. Clinical prognostic scores such as the PESI and hemodynamic status at the time of the PE may be useful predictors of mortality; however, even when hypotensive patients were excluded from analysis, the presence of a right heart thrombus remained an independent predictor of mortality. For example, a three-fold increase in mortality was noted in a subgroup of submassive or intermediate-risk PE patients with concomitant CIT [1,5,6].

The potential benefit of management beyond systemic heparin for CIT was suggested in a 2002 meta-analysis of 177 patients with CIT. This study demonstrated the mortality rates associated with no therapy, anticoagulation, surgical embolectomy, and thrombolysis were 100%, 28.6%, 23.8%, and 11.3%, respectively, though the analysis did not specify whether the mortality rates reported were in-hospital, within 28 days, or a mix of the two [7]. While there are no randomized controlled trials to help establish guidelines for the management of acute PE with concomitant CIT, much of the literature supports the superiority of reperfusion therapy, namely, thrombolysis or thrombectomy, over anticoagulation alone in patients with both submassive and massive PE. Studies on submassive PE management have shown thrombolysis to reduce rates of hemodynamic decompensation, albeit with a known added risk of major intracranial hemorrhage and stroke [3,6,8]. The current literature lacks any definitive comparison between thrombectomy and thrombolysis. The risks of thrombolytic therapy speak to the potential need for a separate non-thrombolytic intervention for patients with submassive PE and concomitant CIT.

In the case of our patient, he was hemodynamically stable but had high predicted mortality with an elevated PESI score and identified CIT by echo. He underwent a mechanical thrombectomy using an Inari FlowTriever on day two after diagnosis of PE and CIT. The FlowTriever is a Food and Drug Administration (FDA)-approved device (2021) for the treatment of CITs and has been used successfully in the literature [9,10]. Other methods of mechanical thrombectomy such as the Angiovac device have also been reported with mixed patient outcomes. Compared to other embolectomy systems, the FlowTriever possesses the advantage of being less rigid, more mobile, and does not require extracorporeal filtration or cardiopulmonary bypass during use [2,3,11,12]. In any CIT embolectomy case, a risk of embolization during retrieval or potential hemodynamic compromise exists; however, this case supports a management strategy that safely removed a large mobile right heart thrombus without general anesthesia while also avoiding the risks associated with thrombolysis or cardiothoracic surgery. 

A final note of interest concerns how the character of a CIT may affect the standard of care. Available studies lack detailed information on the morphology and mobility of the right heart thrombi. Because diagnostic workup is generally not controlled, patients referred for echocardiography tended to present with more hemodynamic compromise compared to the patients who did not receive imaging [1,4,7]. Considering the high number of patients who were excluded from analyses due to lack of echocardiography, it is possible that CITs have a higher than previously anticipated incidence. Furthermore, the CIT in our patient is unique and does not match the typical Type A or B categorizations and is grossly different from the free-floating, serpentine thrombi that have been reported in cases that used mechanical thrombectomy [9,12,13]. The significance of this finding is yet to be determined but offers an area to be investigated.

## Conclusions

There is no established management standard for patients with acute PE and CIT due to the lack of randomized trials assessing the efficacy of anticoagulation alone compared to anticoagulation with reperfusion therapies. While much of the literature supports the use of reperfusion therapy, without a standard guideline for care, each case of CIT necessitates an individual assessment of patient and clot characteristics. Ultimately, our case demonstrates the successful usage of the FlowTriever device in a patient with a unique tricuspid-adhered CIT and concomitant acute submassive PE and supports percutaneous intervention with flexible mechanical thrombectomy in patients with intermediate-high risk PE and a right heart thrombus on echocardiogram, with the benefit of avoiding risks associated with thrombolysis.
